# Real-world performance of the AI diagnostic system IDx-DR in the diagnosis of diabetic retinopathy and its main confounders

**DOI:** 10.1038/s41598-026-36970-9

**Published:** 2026-01-29

**Authors:** Elisabeth Hunfeld, Allam Tayar, Sebastian Paul, Broder Poschkamp, Rico Großjohann, Eva Morawiec-Kisiel, Beathe Bohl, Johanna M. Pfeil, Martin Busch, Merlin Dähmcke, Tara Brauckmann, Sonja Eilts, Marie-Christine Bründer, Milena Grundel, Bastian Grundel, Frank Tost, Jana Kuhn, Jörg Reindel, Petra Augstein, Wolfgang Kerner, Andreas Stahl

**Affiliations:** 1 Department of Ophthalmology, University Medical Center Greifswald, Ferdinand-Sauerbruch-Straße, 17475 Greifswald, Germany; 2 Hospital for Diabetes and Metabolic Diseases Karlsburg, Greifswalder Str. 11, 17495 Karlsburg, Germany

**Keywords:** Diabetic retinopathy, AI-based diagnostics, IDx-DR, Retinal Imaging, Retinal diseases, Medical imaging

## Abstract

The escalating prevalence of diabetes mellitus (DM) emphasizes the critical need for early detection of diabetic retinopathy (DR). This study assesses the performance of the autonomous AI-based diagnostic system IDx-DR in detecting DR and its associated confounders in a real-world clinical setting. This prospective cross-sectional study involved 875 diabetic patients with a mean age of 52 years (range: 8–92). Retinal images were captured by trained assistants. IDx-DR results were compared with mydriatic fundus examination (gold standard) and Ophthalmologists’ image analysis. Factors impacting image acquisition or analyzability were examined. Among all patients, 10.5% yielded no image in miosis, and 26.1% were unanalyzable by IDx-DR. Confounders affecting image acquisition were examiner, pupil size, patient age and patients’ visual acuity. When good quality images were achieved, IDx-DR performed well, particularly in detection of severe DR (sensitivity 94.4%; specificity 90.5%). IDx-DR results exactly matched Ophthalmologists’ mydriatic fundoscopy gradings in 54.2% if images of sufficient quality were obtainable. Undergrading of DR severity by IDx-DR was rare (4.8%). IDx-DR shows promise in detecting DR, especially in resource-limited settings and in detecting severe DR. One remaining challenge is good image acquisition in miotic patients.

**Supplementary Information:**

The online version contains supplementary material available at 10.1038/s41598-026-36970-9.

## Introduction

With growing numbers of patients worldwide, diabetes mellitus (DM) is a disease that will continue to be of rising concern in the future. In Germany, a prevalence estimate predicted an increase of cases of type 2 diabetes from 6.9 million in 2015 to 8.3 million (+ 21%) by 2040 ^1^. About one third of all diabetic patients have a diabetic retinopathy (DR) ^2^.Screening and early treatment of retinopathy can reduce the risk of blindness due to DR by approximately 56% ^3^. Early detection of DR would therefore be desirable. The autonomous artificial intelligence (AI)-based diagnostic system IDx-DR (Digital Diagnostics, Coralville, IA, USA) enables such an approach by cloud-based analysis of ocular fundus images taken by trained staff and automatically classifying them into no, mild, moderate, or severe (vision-threatening) DR. IDx-DR was the first AI-based diagnostic system using deep learning (which is also used by other medical specialties) with FDA approval in the US and with CE marking in Europe as a Class IIa medical device for autonomous detection of diabetic retinopathy ^4,5^.

In this project, we used the IDx-DR screening system to examine patients admitted for inpatient treatment at a tertiary diabetes center in Karlsburg, Germany. Our research aimed to contribute to the understanding of the real-world application of AI-based diagnostic tools for DR in a clinical setting. The study analyzes a relatively large patient population across a wide age range and includes all present diabetes subtypes. All our analyses were done with non-dilated pupils as this best represents a true screening setting that must be able to function in the absence of an Ophthalmologist and thus should avoid any medical intervention that can potentially put patients at risk, as pupil dilation might do in patients at risk of angle closure. The results of our study show the potential as well as the limitations of an AI-based screening tool for DR and identify some of the parameters associated with obtaining reliable screening results.

## Materials and methods

### Study population

The presented study is a prospective cross-sectional study. The target population included patients with DM admitted for inpatient treatment at the Karlsburg Diabetes Hospital between February 2020 and November 2021. The study was conducted in accordance with the Declaration of Helsinki and ethical approval from the Ethics Committee of the University Medical Center Greifswald (BB 025/20) had been obtained. The inclusion criterion for this study was the presence of DM, no exclusion criteria were applied except for patients declining participation in the study procedure. Written informed consent was obtained from all patients or legal representatives prior to study inclusion.

### Study procedures

**Fig. 1:**
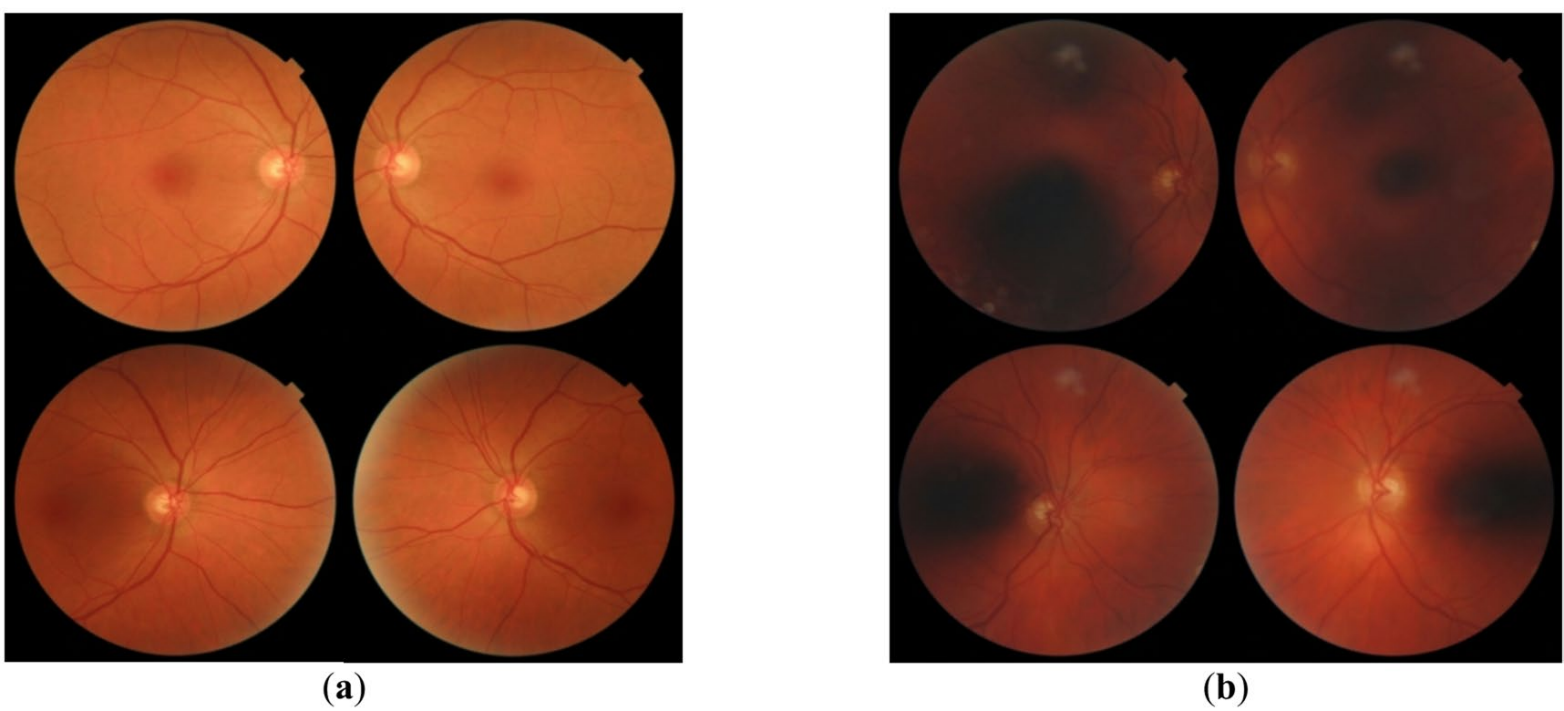
(**a**) Exemplary images of a patient whom the IDx-DR artificial intelligence system scored as having no DR.; (**b**) Exemplary images of a patient whom the IDx-DR artificial intelligence system graded as “Test quality insufficient” (means not analyzable).

On the first appointment, demographic information (age, gender, body mass index etc.) as well as information regarding medical history (diabetes duration, duration of insulin use, underlying eye conditions, blood pressure, etc.) were documented for each patient. Also, visual acuity (VA) (distant and near vision (Snellen chart), intraocular pressure [mmHg] and refraction [dpt]) were assessed. Subsequently, four retinal images (two per eye, one centered on the optic disc, one on the macula; starting with the right eye), were acquired by one of four trained technical assistants (later referred to as examiners) using a Topcon TRC-NW400 camera or the Zeiss VISUCAM 500 camera. The patient takes a frontal position in front of the non-mydriatic retinal camera in a darkened examination room. Images (Fig. 1) were transmitted to the IDx-DR-software, which automatically evaluated the images resulting in one diagnosis per patient (no, mild, moderate or severe DR), primarily relying on the condition of the more severely affected eye ^6,7^.

In case of insufficient image quality, the output of the IDx-DR was “insufficient test quality”. Once the images had been transmitted to the AI system and image quality was assessed as insufficient by the system, retaking of images was not allowed according to our study protocol to enable a work-flow that would be feasible for a real-life clinical scenario. In addition to IDx-DR grading, all images were stored and at a later timepoint were graded in a blinded manner by one of four trained Ophthalmologists^8^. The Ophthalmologists consisted of specialists from the University Eye Hospital Greifswald with at least 7 years of professional experience. Additionally, two days after image acquisition and IDx-DR grading, all patients were seen by one of four trained Ophthalmologists and obtained a full Ophthalmological assessment including funduscopic examination of both eyes in mydriasis. This represents our gold standard, as this reflects the clinical routine at our center and aligns with the German National Health Care Guideline (NVL), which continues to recommend this method as the standard for diabetic retinopathy screening ^9^. Ophthalmologists performing the image analysis or the funduscopic examination grouped patients into the same categories as IDx-DR (no, mild, moderate or severe DR). No diabetic retinopathy corresponds to level 10 of the ETDRS-grading, mild equals level 20, moderate corresponds to the levels 35, 43, and 47 and severe DR corresponds to ETDRS levels 53 a-e, 61, 65, 71, 75, 81, and 85 ^10,11^. In summary, we obtained five results for each patient: 1) IDx-DR diagnosis on a patient level, 2/3) result of mydriatic ophthalmological fundus examination for each eye separately and 4/5) ophthalmological image analysis for each eye separately. For the comparison of the mydriatic fundus examination or the expert image analysis to IDx-DR results, the eye with the higher degree of DR on mydriatic fundoscopy or image analysis was used as IDx-DR always only returns DR severity at the patient level, rather than eye level. A subgroup of patients (N = 141) who were included in the later phase of the study also received measurements of pupil size using the plusoptixA12c binocular autorefractometer (Plusoptix GmbH, Nürnberg, Germany), as pupil size was added as an additional parameter during the course of the study due to its potential relevance for image quality and analyzability.

### Data analysis and statistics

Statistical analysis including cleaning and formatting of raw data, was done with SPSS (IBM SPSS Statistics V.27, IBM Corporation, Armonk, NY, USA), GraphPad Prism (Prism 9, GraphPad Software, Inc., La Jolla, USA), Python 3.10 (Python Software Foundation) and Microsoft Excel (Microsoft Excel V.16.57, Redmond, WA, USA). Mean values, standard deviations (SD), sensitivity and specificity of IDx-DR results in dependence of the funduscopic diagnosis were calculated. In addition, we determined the Cohen´s kappa coefficient (κ) interpreting the strength of agreement according to Landis and Koch ^12^. Quantitative variables are summarized by mean ± SD, multiple comparisons were performed with ANOVA and Kruskal–Wallis tests depending on type of data distribution. Nominally scaled variables were analyzed with a two-sided Chi-square test, or a two-sided Fisher´s exact test respectively. Outliers in continuous variables were removed using the ROUT method (Q = 5%), which, while originally developed for nonlinear regression, has been increasingly applied in biomedical research for general outlier detection outside regression models due to its robustness and false discovery rate control ^13^. Asterisks indicate the level of significance as followed: *, **, *** for the *p*-values ≤ 0.05, ≤ 0.01, ≤ 0.001.

We analyzed potential influencing factors on image acquisition and analyzability in a two-step approach. In this study, the term confounder is not used in a strict statistical sense, but rather in a broader, application-oriented context. First, we descriptively screened 141 candidate factors (pre-existing conditions, demographics, ocular characteristics) by comparing the proportions of (i) no image obtained and (ii) images rated not analyzable across factor levels (e.g., cataract present vs absent). No inferential statistical tests were performed in Step 1, and no *p*-values were generated or interpreted at this stage. Second, we conducted confirmatory tests on 10 clinically relevant factors per outcome. Multiplicity was controlled using Bonferroni method with family-specific α correction (per-test α* = 0.005).

## Results

### Study population

For this study, 878 diabetic patients were enrolled. Three were discharged before all study examinations took place and were thus excluded from the study (N = 875). 15 Patients (1.7%) had four retinal images taken and underwent IDx-DR analysis but were discharged before the mydriatic funduscopic examination could take place. In addition, no images could be taken in three other patients (0.3%) who already underwent medical history and visual acuity testing and were also discharged before the mydriatic funduscopic examination could take place. The statistical analysis included 1750 eyes from 875 patients (502 male, 373 female) with a mean age of 52 years ± 18.1 (range: 8–92 years). The study consisted of 433 patients with type 1 diabetes (49.5%), 430 with type 2 diabetes (49.1%) and 12 with other types of diabetes (1.4%) (Table 1).

**Table 1:** Study population.

Variables	Patients (n = 875)
Sex (Male/Female)	502/373
Age (Mean ± SD) Minimum Age Maximum Age	52 ± 18.1 8 92
Diabetes Type Type 1 Diabetes Type 2 Diabetes Other Types of Diabetes	433 430 12

For a total of 92 patients (10.5%) the examiners were unable to obtain an image and 228 patients (26.1%) were assessed as not analyzable by IDx-DR. In 555 patients, images could be classified by IDx-DR (63.4%), of which 229 were diagnosed as no DR (41.3%), 174 as mild DR (31.4%), 69 as moderate DR (12.4%) and 83 as severe DR (15.0%) by the IDx-DR algorithm.

### Performance of IDx-DR

**Fig. 2:**
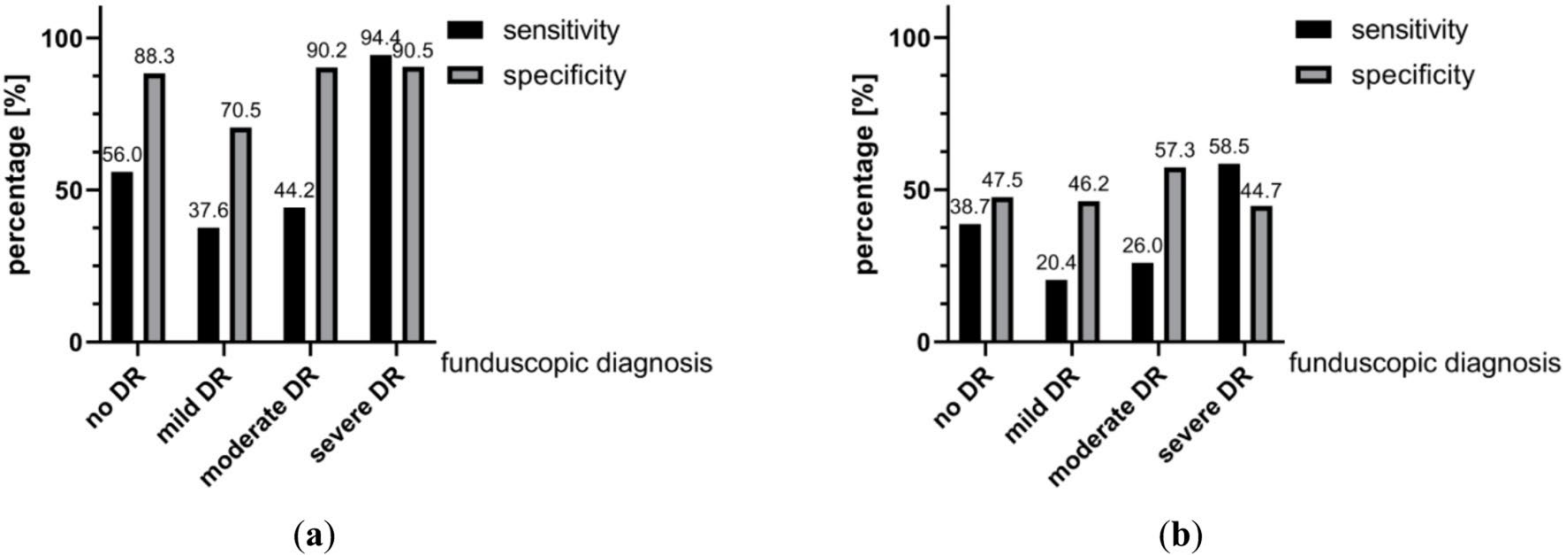
(**a**) Sensitivity and specificity of diagnosed IDx-DR results in dependence on the funduscopic diagnosis (gold standard). (**b**) Sensitivity and specificity of all IDx-DR results (including not analyzable and no picture) in dependence on the funduscopic diagnosis (gold standard).

We analyzed the performance of the IDx-DR autonomous AI-based diagnostic system by calculating its sensitivity and specificity compared with our gold standard, the binocular fundus examination by an Ophthalmologist (Fig. 2).

In Fig. 2a we only included results of patients with analyzable pictures by IDx-DR (no DR, mild DR, moderate DR, severe DR). When including also the patients with no picture or the IDx-DR result “not analyzable”, a reduction in all sensitivity and specificity values was observed (Fig. 2b). The highest sensitivity (94.4%) and specificity (90.5%) were achieved by IDx-DR in patients with severe DR after excluding patients with no image and images that were rated not analyzable by IDx-DR (Fig. 2a).

A direct comparison of the three diagnostic methods is displayed in Table 2 and 3.

**Table 2:**
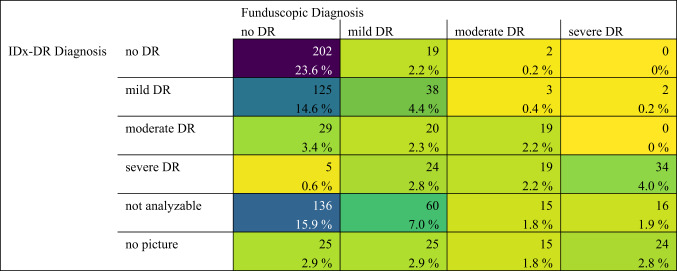
Confusion matrix comparing IDx-DR diagnosis to gold standard Ophthalmic diagnosis using dilated fundoscopy.

**Table 3:**
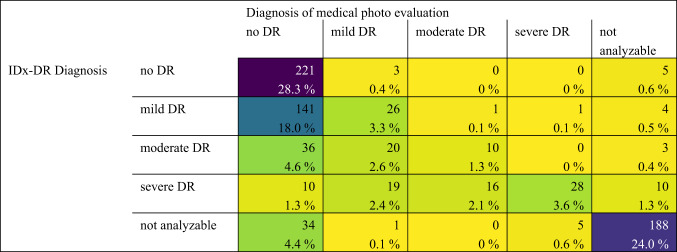
Confusion matrix comparing IDx-DR diagnosis to Ophthalmic diagnosis using fundus images (the same ones as used by the IDx-DR algorithm).

Corresponding scatter plots are provided in supplementary Figs. 1 and 2, including a scatterplot comparing funduscopic and photo-based diagnoses. When comparing IDx-DR results with the gold standard of mydriatic funduscopic examination (Table 2), one observes an absolute match in 34.2% if all patients are included who underwent a funduscopic examination (even ones without image or insufficient quality where IDx-DR did not provide a grading). When considering only patients that had images acquired in sufficient quality for an IDx-DR diagnosis (i.e., excluding patients with unanalyzable or no image) an exact match between IDx-DR and mydriatic fundoscopy was achieved in 54.2% of cases.

In 41.0% of analyzable patients, the IDx-DR system overestimated the severity of diabetic retinopathy and in only 4.8% of analyzable patients IDx-DR underestimated the severity of DR. 26.5% of all patients who underwent a funduscopic examination were graded “not analyzable” by IDx-DR and in 10.4% of patients no image could be obtained.

When comparing IDx-DR with the Ophthalmologist’s diagnosis based on fundus image evaluation (Table 3), IDx-DR matched the image-based Ophthalmologist’s diagnosis in 60.5% of all patients with an image available (including the ones where IDx could not yield a diagnosis due to insufficient image quality). If only patients are considered for whom both IDx-DR and the examining Ophthalmologist graded the images as analyzable, the agreement was 51.4%. 43.6% of patients who had analyzable images for both IDx-DR and human graders were overestimated in their DR severity by IDx-DR, and only 0.9% of these were underestimated by IDx-DR. 5.1% of all available images were considered analyzable by Ophthalmologists while graded as not analyzable by IDx-DR and vice versa 2.8% of all images were considered not analyzable by Ophthalmologists while considered analyzable by IDx-DR.

In 26 patients who underwent successful IDx-DR analyses and medical funduscopic examination, IDx-DR diagnosed a milder form of DR compared to the funduscopic diagnosis (4,8%, Table 2). It has to be taken in mind, that for this comparison the eye with more diabetic changes upon fundoscopy was used (IDx-DR only reports on a patient level, not on eye level). In 10 of these 26 cases (38.5%), the IDx diagnosis concurred with the funduscopic diagnosis of the milder affected eye.

Comparing IDx-DR diagnosis with mydriatic fundoscopy across all patients (including the ones without image and the ones with images considered not analyzable by IDx-DR) yielded a Cohen´s kappa coefficient of 0.16 (*p*-value < 0.001), which can be graded as “slight agreement” ^12^. If only patients for whom images were available and analyzable for IDx-DR were included, the Cohen´s kappa coefficient for comparison of IDx-DR to the gold standard of mydriatic fundoscopy was 0.28 (*p*-value < 0.001), which corresponds to a “fair agreement” ^12^. Comparing IDx-DR with the Ophthalmologist’s image evaluation across all patients (including the ones without image and the ones with images considered not analyzable by IDx-DR) yielded a Cohen´s kappa coefficient of 0.54 (*p*-value < 0.001; “moderate agreement”) ^12^. If only patients that were analyzable for both IDx-DR and the human grader are considered, the Cohen´s kappa coefficient for comparison of IDx-DR to the Ophthalmologists’ image-based diagnosis was 0.25 (*p*-value < 0.001; “fair agreement”) ^12^.

### Examiner effects

**Fig. 3:**
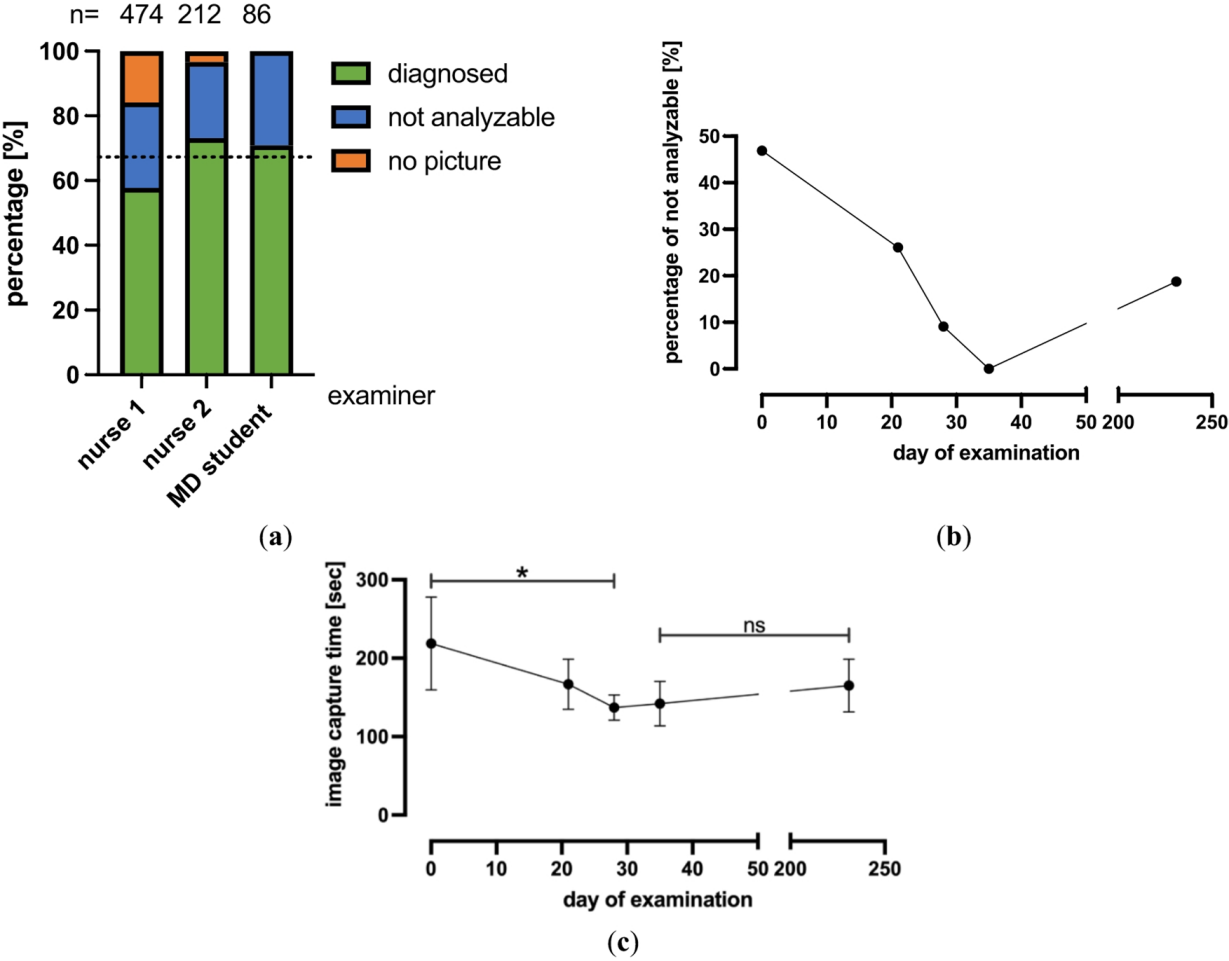
(**a**) Results of the IDx-DR analysis by examiner. Dotted line represents the average percentage of diagnoseable exams (67,3%). (**b**) Percentage of not analyzable pictures by IDx-DR in relation to the MD student´s day of examination. (**c**) Mean value and SD of the time required for image acquisition in relation to the MD student´s day of examination. * Indicates *p* ≤ 0.05 (Dunnett’s T3 multiple comparison test).

After evaluating the baseline performance of IDx-DR in our cohort, we aimed to identify the main confounders affecting grading results and started with analyzing the effect of the examiner. First, we analyzed the number of examinations done by each of our four examiners. As one examiner assessed less than ten patients, this examiner was excluded from all consequent analyses and only the remaining three examiners were compared (Fig. 3a).

From all patients examined by nurse 1 (N = 474), 125 (26.4%) were not analyzable by IDx-DR and 75 (15.8%) yielded no image. From all patients examined by nurse 2 (N = 212), 50 (23.6%) were not analyzable by IDx-DR and 7 (3.3%) yielded no image (all in miosis). From all patients examined by the MD student (N = 86), 25 (26.1%) were not analyzable by IDx-DR and there were no patients that yielded no image. All patients from all examiners were examined in normal pupillary condition, no dilating eye drops were given.

As a next step, we investigated whether there is a learning curve for image acquisition. For this purpose, we analyzed the performance of the MD student regarding the percentage of not analyzable images acquired (Fig. 3b) and the time needed for image acquisition (Fig. 3c). The percentage of images that were not analyzable by IDx-DR decreased over time from 46.9% on the first day of using the method to 0% on day 4 of training. After an extended period of no practice due to COVID-19 limiting access to outpatient services (196 days), the percentage of not analyzable images increased again to 18.8%. The time needed to acquire a complete set of images per patient (in total 4 images, one per eye centered on the optic nerve head and one on the fovea) showed a similar pattern (from 219 ± 59 s on day 1, to 137 ± 16 s and a final value of 165 ± 34 s on day 231; Fig. 3c).

### Most relevant confounders for image acquisition and picture-analyzability

To take an unbiased look at the factors that may impact image acquisition or analyzability by IDx-DR, we compared 141 pre-existing conditions, demographic characteristics or physical features (e.g.: pupil size and BMI) that were present in at least 10 patients of our cohort. For each potential confounder, the proportion of missing images and missing analyses was calculated. Table 4 displays the 10 items with the highest percentages of patients with no image or an image that was not analyzable by IDx-DR.

**Table 4:** Top 10 confounders (total 141) associated with inability to acquire or analyze image by IDx-DR. (DME = diabetic macular edema, DR = diabetic retinopathy, VEGF = vascular endothelial growth factor, BCVA = best corrected visual acuity).

Percentages of no images (confounder n > 10)					Percentages of not analyzable images (confounder n > 10)				
	Item	n	Percentage	*P*-value		Item	n	Percentage	*P*-value
1	Left eye DME documented	16	50	< 0.0001^a^	1	Right eye impaired fundus view (cataract)	18	61	< 0.0002^b^
2	Left pupil size first quartile (< 3.1 mm)	37	41	< 0.0001^c^	2	Left eye impaired fundus view (cataract)	20	60	< 0.0001^b^
3	Zeiss camera used	47	40	< 0.0001^a^	3	Right pupil size second quartile (3.0–3.7 mm)	29	52	< 0.0001^c^
4	Right eye DME documented	21	38	< 0.0001^a^	4	Left pupil size second quartile (3.1–3.8 mm)	35	43	< 0.0001^c^
	Right pupil size first quartile (< 3.0 mm)	42	38	< 0.0001^c^					
5	Left eye severe DR diagnosed by funduscopy	63	37	< 0.0001^a^	5	Right eye intravitreal anti-VEGF treatment documented	13	38	*0.1435 (ns)*^a^
6	Left eye retinal laser treatment documented	97	34	< 0.0001^a^	6	Age fourth quartile (64.5–92.0 years)	219	38	< 0.0001^d^
7	Right eye retinal laser treatment documented	101	34	< 0.0001^a^	7	Left eye DME documented	16	38	0.0093 (*ns*)^b^
8	Right eye severe DR diagnosed by funduscopy	68	32	*0.0659 (ns)*^b^	8	Age third quartile (57.0–64.5 years)	195	36	< 0.0001^d^
9	Left eye impaired fundus view (cataract)	20	25	0.0293 (*ns*)^a^	9	Left eye BCVA first quartile (< 0.63 BCVA decimal)	220	36	< 0.0001^d^
10	Right eye intravitreal anti-VEGF treatment documented	13	23	*0.1483 (ns)*^b^	10	Right eye BCVA first quartile (< 0.63 BCVA decimal)	222	35	< 0.0001^d^

The highest percentage with 50% of patients yielding no full image set for analysis was seen in patients with DME in the left eye. The highest percentage (61%) of not analyzable pictures was found in the group of patients with documented impaired view of the retina in the right eye due to cataract.

**Fig. 4:**
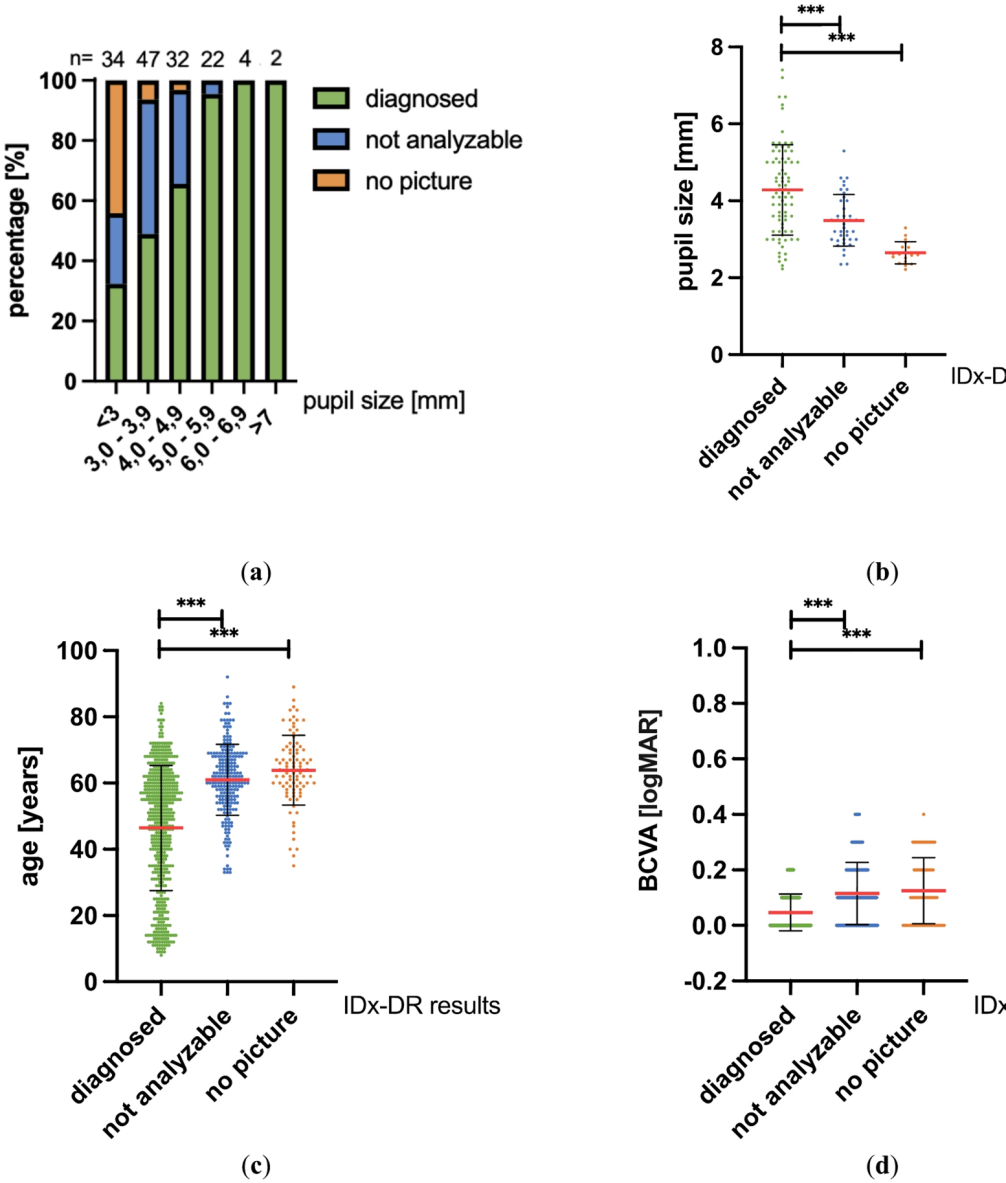
(**a**) Effect of pupil size on image acquisition and analyzability. For each person, the narrower available pupil size was used for analysis. Total number of pupil sizes = 141. (**b**) Mean + /- SD values for pupil sizes from patients who were diagnosed by IDx-DR, were not analyzable or had no image. *** indicates *p* ≤ 0.001 (Dunnett’s T3 multiple comparisons test) (**c**) Mean + /- SD values for patient age from patients who were diagnosed by IDx-DR, were not analyzable or had no image. *** indicates *p* ≤ 0.001 (Dunnett’s T3 multiple comparisons test) **(d)** Mean + /- SD values for BCVA from patients who were diagnosed by IDx-DR, were not analyzable or had no image. *** indicates *p* ≤ 0.001 (Dunnett’s T3 multiple comparisons test).

Figure 4 displays the effect of pupil size, patient age and best corrected visual acuity (BCVA) on image acquisition and analyzability.

In this context, the smaller pupil of a patient was utilized, resulting in no differentiation between the right or left pupil of a patient compared to the data presented in Table 4 (the cut-off limits vary in the course of this). Smaller pupil sizes resulted in lower percentages of diagnoseable images by IDx-DR, yielding only 32% analyzable patients in the group with pupil size < 3 mm (Fig. 4a). Mean pupil size of patients with an IDx-DR diagnosis was 4.28 mm, mean pupil size of non-analyzable patients was 3.49 mm, and mean pupil size of patients without a full image set was 2.65 mm (diagnosed vs. not analyzable p = 0.0001; diagnosed vs. no picture p =  < 0.0001; Fig. 4b). Patient age also had a statistically significant impact on image acquisition and analyzability (Fig. 4c). The group of patients for whom IDx-DR was able to issue a diagnosis was the youngest group, those with a mean age of 46.4 years, while patients who could not be analyzed or had no full image set had a mean age of 61.0 and 63.9 years respectively. We also observed that BCVA had a statistically significant impact on image acquisition and analyzability by IDx-DR. Mean BCVA for diagnoseable patients was 0.047 logMAR while patients who could not be analyzed or had no full image set had BCVA of 0.116 and 0.155 logMAR respectively (Fig. 4d).

## Discussion

Our study found an overall good performance of IDx-DR as a pre-examination tool in the detection of diabetic retinopathy in patients with diabetes mellitus for whom analyzable images can be provided (Fig. 2). Most importantly, in cases of misalignment with the gold standard funduscopic examination, IDx-DR more often overestimates than underestimates disease severity – a type of error that does not put the patient at danger. Taking a closer look at the performance of IDx-DR, sensitivity and specificity were highest for severe DR and lower for mild and moderate DR (Fig. 2a). This finding is consistent with other study results ^7,14^. Especially in the early stages of diabetic retinopathy, the subdivision between mild and moderate stages is challenging due to subtle and overlapping lesion patterns. Even among human expert graders, only moderate interobserver agreement has been found in previous studies ^15–17^.

Underestimation of DR severity, meaning a milder classification of DR by IDx-DR compared to the diagnosis made based on gold standard mydriatic fundoscopy, occurred rarely (4.8% of all patients for whom images were available and analyzable, Table 2). More often, IDx-DR overestimated the severity of DR compared to the physician (41.0% for all patients for whom images were available and analyzable). This overall “careful” approach to DR grading by IDx-DR is sensible for a device used as a DR screening device where it is important to not miss severe disease stages ^7,18^. On the other hand, this can lead to a potentially unnecessary presentation to the Ophthalmologist, or create an uncertainty for patients when more severe disease stages are diagnosed than are later confirmed by the examining Ophthalmologist ^19^. Ideally, IDx-DR should yield the exact same diagnosis as the gold standard fundoscopy exam. This was the case in 54.2% of patients when excluding those with unanalyzable result, no image or no mydriatic funduscopic examination (Table 2). This proportion appears limited and likely reflects, among other factors, known challenges in exact grading agreement, particularly between mild and moderate DR, even among specialists ^15–17^. To address this, we calculated additional sensitivity and specificity values for broader diagnostic categories (mild-severe DR, moderate-severe DR). The analyses showed increased sensitivity in both groupings compared to evaluating mild and moderate stages individually. This improvement is likely due to the reduced complexity and ambiguity introduced by grouping adjacent DR stages into broader categories, which helps minimize misclassification near category boundaries. These results are presented in supplementary Fig. 3a and b. Regarding the patients for whom IDx-DR underestimated disease severity, one has to take into account that IDx-DR reports on a patient level, not eye level and results may differ when comparing them to funduscopic grading of the more severely affected eye (as was done in our funduscopic analysis). Furthermore, in 88,5% of patients for whom IDx-DR underestimated disease severity compared to the gold standard of funduscopic examination, the physician’s image analysis also yielded a milder form of DR compared to the funduscopic grading suggesting that some diabetic changes leading to a more severe diagnosis may lie outside the scope of the acquired images. It should also be noted that the large majority of these underestimated cases where in the region of no DR / mild / moderate DR. Only in 2 cases did IDx-DR underestimate disease severity in patients who were diagnosed with severe DR upon gold standard fundoscopy.

Comparing IDx-DR with the ophthalmologist’s image evaluation in patients with analyzable images for both yielded a Cohen’s kappa coefficient of 0.25 (*p*-value < 0.001), indicating fair agreement. It appears that the current accuracy of the model is limited and should continue to be supervised by a physician, especially with regard to false positive patients. (30.5% of all patients with no DR in the funduscopic examination received a positive diagnosis (mild-severe) from IDx-DR, see Table 2).

In contrast to previous studies, we also calculated sensitivity and specificity of IDx-DR including non-analyzable patients and patients with no images ^18,20,21^. The inclusion of these patients leads, as expected, to a significant reduction in both values, in contrast to analyses that only take into account patients with analyzable image sets. This reduction highlights a key limitation for screening tools, as sensitivity is critical to detect true positives. Our findings emphasize the need to improve image acquisition protocols to enhance the rate of analyzable images and maintain diagnostic accuracy in routine practice. We could detect several pre-existing ophthalmological conditions such as DME and cataract that affect the likelihood with which analyzable images can be obtained. In addition, we found that pupil size affects the ability to acquire analyzable images. This can be overcome by dilating the pupil as was done in the studies by Roser et al. or Mehra et al., as long as care is taken that appropriate measures are in place to deal with possible complications like acute narrow-angle glaucoma ^22,23^. It should, however, be kept in mind that acute angle-closure glaucoma is a rare complication, as demonstrated by the Rotterdam study involving 6760 participants, where it occurred in only two individuals (0.03%) aged 55 and over ^24^. As previously found in other studies, we were able to confirm that with increasing age image acquisition and analyzability by IDx-DR becomes less successful ^23^. This is particularly difficult since, with age, pupil width decreases and lens opacities increase ^25,26^. In addition to pupil size and patient age, our study identified low visual acuity as one aspect that has a negative impact on image acquisition and analyzability.

Our study also identified the examiner as having an effect on image acquisition and analyzability. Correct training and sufficient time for image acquisition are likely to be key factors for good image acquisition. The study of Goldstein et al. demonstrated the importance of trained staff and on-site workflow for successful AI implementation into clinical practice ^27^. This notion is further confirmed by the fact that we found significant improvement over time in the rate of analyzable images obtained with dedicated training (Fig. 3c). We acknowledge that the rate of analyzable images would have been higher if examiners had been allowed to go back to retake images after receiving negative feedback from the IDx Software regarding analyzability of the submitted images. However, in an attempt to allow for a more accurate comparison of examiner quality under consistent conditions and in order to simulate a real-life scenario in a busy office setting with constraints regarding time and workflow, we opted to exclude the possibility of retaking images.

Our study has several limitations. The study population remains relatively small compared to a population-wide screening and is biased in terms of disease severity, as it consists of patients already referred to a diabetes clinic. Additionally, since we did not collect data on ethnicity in our study, we cannot comment on generalizability of the AI performance across different ethnic groups. These limitations underscore the need for larger and more diverse study settings to better understand the performance and applicability of AI-based systems in broad clinical practice. Moreover, pupil size was not measured in all patients, so the number of values for this analysis was smaller compared to other parameters investigated. Having four different Ophthalmologists doing the gold standard mydriatic fundus exams reduces individual bias but bears the risk of inter-individual grading variability ^15,16,28,29^. We did not have a second (or third) grader who confirmed (or challenged) funduscopic or image-based diagnoses made by the Ophthalmologists as would have been the case in a reading-center based setting. Regarding the analysis of confounders, only univariate analyses were performed. We acknowledge that a multivariate approach could more effectively account for interrelated variables and represents an important direction for future research.

In summary, IDx-DR displayed a high sensitivity and specificity in the detection of severe DR in our study in a real-life clinical setting – under the condition that appropriate image sets were obtainable. The Diabetes Center in Karlsburg does not have an Ophthalmologist permanently on site. Ophthalmological assessments are only offered once a week. In this context, AI-based screening could help prioritize patients with suspected referable DR, thereby reducing unnecessary specialist exams and associated costs while optimizing resource allocation ^30^. Nevertheless, its use as a valid screening tool without strict monitoring remains complicated at present. Further research is warranted, particularly in terms of exploring novel approaches or modifications to image acquisition protocols to improve the rate of obtainable and analyzable retinal images. For instance, investigating the application of pupil dilating eye drops or alternative techniques capable of capturing high-quality images and analyze them (for example as a multi-layer stacked ensemble) in patients with challenging image acquisition settings such as small pupil size, lens opacities, or low visual acuity, as identified in the current study, is imperative ^31^. Looking into the future, it will be crucial to conduct external validation studies across diverse clinical settings and populations to evaluate the generalizability and external validity of AI-based DR screening systems like IDx-DR. This could involve collaboration with multiple healthcare facilities to validate the system´s performance across different geographic regions, ethnicities, and healthcare infrastructures, as well as the assessment of one patient by several Ophthalmologists. In these real-world settings, it would also be interesting to analyze how AI based screening systems are accepted by patients ^32^. Additionally, research should explore the impact of examiner training and standardization protocols on image quality and acquisition performance. Assessing the effectiveness of targeted training programs, workflow optimizations, and quality assurance measures is essential to enhance the consistency and reliability of DR screening results in real-world settings.

## Supplementary Information

Below is the link to the electronic supplementary material.

Supplementary Material 1

## Data Availability

The data presented in this study are available on request from the corresponding author. The data are not publicly available due to privacy of the patients.
