# Supplementary material for: Real-world performance of the AI diagnostic system IDx-DR in the diagnosis of diabetic retinopathy and its main confounders
Source: Sci Rep. 2026 Jan 29;16:4349. doi: 10.1038/s41598-026-36970-9 (PMC12864748; doi:10.1038/s41598-026-36970-9)
Supplement: Supplementary file 1 — Supplementary Material 1 [file 41598_2026_36970_MOESM1_ESM.docx]

**Supplementary Materials**


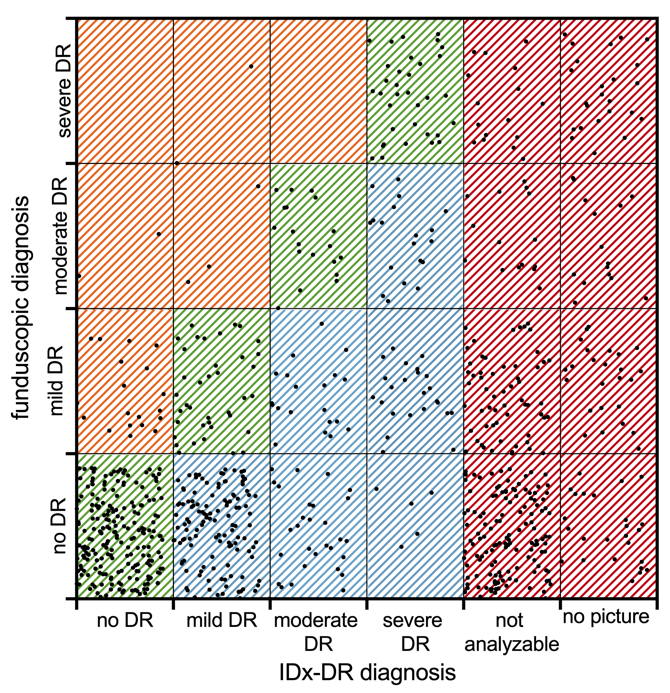

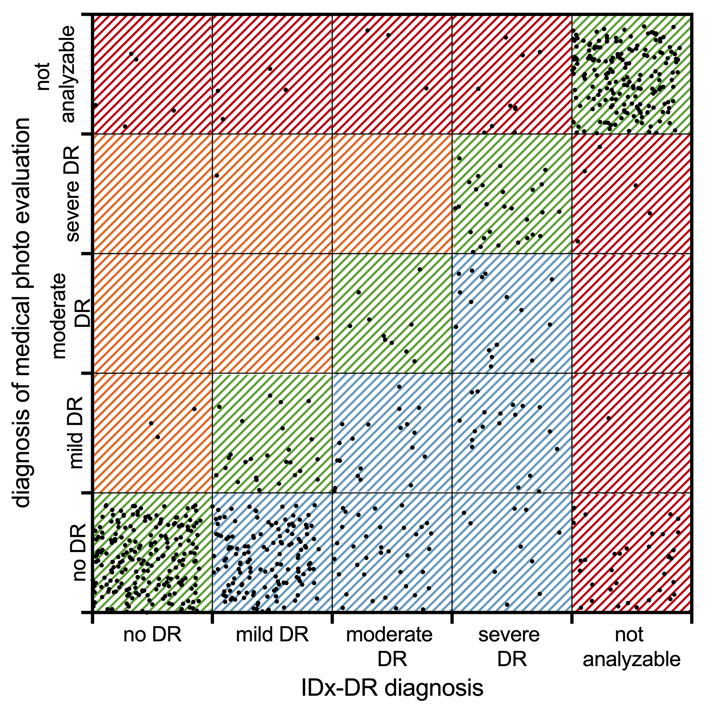


|  |  |
| --- | --- |
| (**a**) | (**b**) |

**Supplementary Figure 1. (a)** Scatterplot comparing IDx-DR diagnosis to gold standard Ophthalmic diagnosis using dilated funduscopy. **(b)** Scatterplot comparing IDx-DR diagnosis to Ophthalmic diagnosis using fundus images (the same ones as used by the IDx-DR algorithm).

**
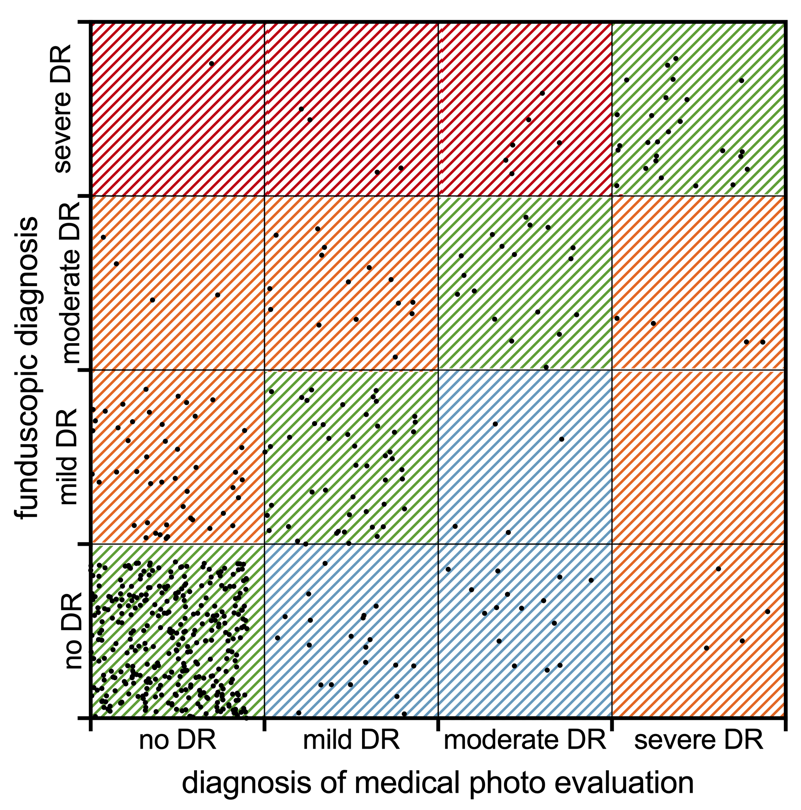
**

**Supplementary Figure 2.** Scatterplot comparing Ophthalmic diagnosis using fundus images (the same ones as used by the IDx-DR algorithm) to gold standard Ophthalmic diagnosis using dilated funduscopy.

**
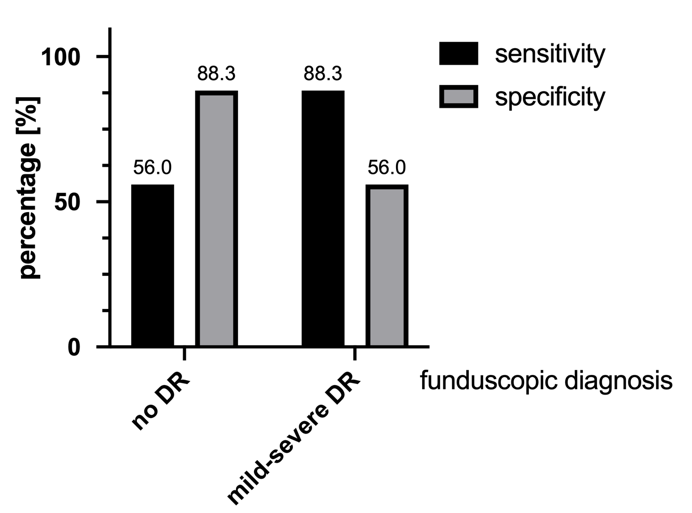

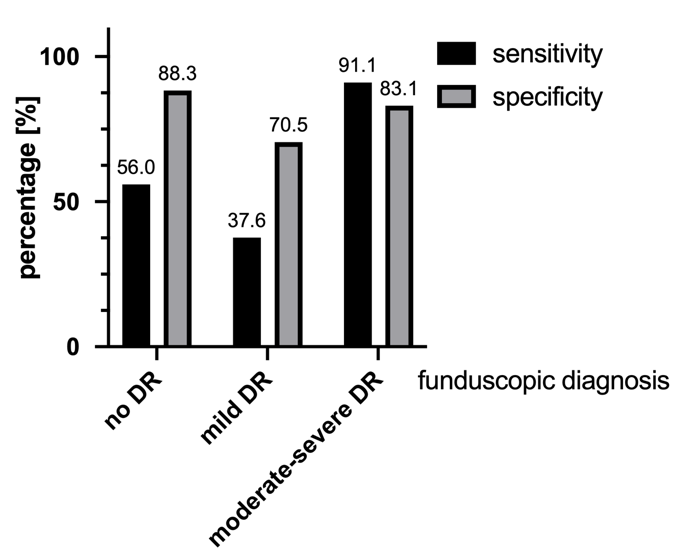
**

| (**a**) | (**b**) |
| --- | --- |

**Supplementary Figure 3. (a)** Sensitivity and specificity of diagnosed IDx-DR results based on funduscopic diagnosis (gold standard) when mild-severe DR is grouped. (**b)** Sensitivity and specificity of diagnosed IDx-DR results in based on funduscopic diagnosis (gold standard) when moderate-severe DR is grouped.
